# Circadian disruption by simulated shift work aggravates periodontitis via orchestrating BMAL1 and GSDMD-mediated pyroptosis

**DOI:** 10.1038/s41368-024-00331-x

**Published:** 2025-02-25

**Authors:** Yazheng Wang, Rui Li, Qingyuan Ye, Dongdong Fei, Xige Zhang, Junling Huang, Tingjie Liu, Jinjin Wang, Qintao Wang

**Affiliations:** 1 State Key Laboratory of Oral & Maxillofacial Reconstruction and Regeneration, National Clinical Research Center for Oral Diseases, Shaanxi International Joint Research Center for Oral Diseases, Department of Periodontology, School of Stomatology, the Fourth Military Medical University, Xi’an, China; 2 Key Laboratory of Shaanxi Province for Craniofacial Precision Medicine Research, Department of Periodontology, College of Stomatology, Xi’an Jiaotong University, Xi’an, China; 3 State Key Laboratory of Oral & Maxillofacial Reconstruction and Regeneration, National Clinical Research Center for Oral Diseases, Shaanxi Key Laboratory of Stomatology, Digital Dentistry Center, School of Stomatology, the Fourth Military Medical University, Xi’an, China; 4 Department of Stomatology, the Seventh Medical Center of PLA General Hospital, Beijing, China

**Keywords:** Periodontitis, Mechanisms of disease

## Abstract

Approximately 20% to 30% of the global workforce is engaged in shift work. As a significant cause of circadian disruption, shift work is closely associated with an increased risk for periodontitis. Nevertheless, how shift work-related circadian disruption functions in periodontitis remains unknown. Herein, we employed a simulated shift work model constructed by controlling the environmental light-dark cycles and revealed that shift work-related circadian disruption exacerbated the progression of experimental periodontitis. RNA sequencing and in vitro experiments indicated that downregulation of the core circadian protein brain and muscle ARNT-like protein 1 (BMAL1) and activation of the Gasdermin D (GSDMD)-mediated pyroptosis were involved in the pathogenesis of that. Mechanically, BMAL1 regulated GSDMD-mediated pyroptosis by suppressing NOD-like receptor protein 3 (NLRP3) inflammasome signaling through modulating nuclear receptor subfamily 1 group D member 1 (NR1D1), and inhibiting *Gsdmd* transcription via directly binding to the E-box elements in its promoter. GSDMD-mediated pyroptosis accelerated periodontitis progression, whereas downregulated BMAL1 under circadian disruption further aggravated periodontal destruction by increasing GSDMD activity. And restoring the level of BMAL1 by circadian recovery and SR8278 injection alleviated simulated shift work-exacerbated periodontitis via lessening GSDMD-mediated pyroptosis. These findings provide new evidence and potential interventional targets for circadian disruption-accelerated periodontitis.

## Introduction

Due to the circadian cycle established by the Earth’s rotation, organisms have developed periodic biological behaviors, physiological functions, and cellular activities to optimize their survival advantages.^1^ In mammals, environmental light signals are processed and projected to the suprachiasmatic nucleus that regulates the timing of the central and peripheral circadian clocks of the body, controlling thermogenesis, metabolism, immunity, and other physiological processes, which is of great significance for maintaining body homeostasis.^2^ Circadian rhythms are primarily regulated by transcriptional-translational feedback loops comprised of core circadian proteins containing brain and muscle ARNT-like protein 1 (BMAL1), circadian locomotor output cycles kaput (CLOCK), and neuronal PAS domain-containing protein 2.^3^ These circadian proteins act as transcriptional regulators, binding to gene promoters and regulating the transcription of clock-controlled genes.^3^ Collectively, the regulation of circadian rhythms relies on both genetic and environmental mechanisms. As a result, circadian disruption can occur when the environmental light-dark cycles are not synchronized with the body’s internal circadian periods.^4^ This can be caused by many factors such as exposure to artificial lighting, transmeridian flights, and shift work.^5^ Recent studies have illuminated that circadian disruption is strongly associated with many diseases with high morbidity rates, including brain disorders, cardiovascular diseases, and diabetes.^6^ However, the effect of circadian disruption on periodontitis is rarely reported.

Periodontitis is a chronic and progressive inflammatory disease with a high prevalence^7^ and severe consequences worldwide.^8,9^ It is a significant public health concern also due to its association with systemic diseases such as cardiovascular disease, diabetes, and adverse pregnancy outcomes.^10^ Despite extensive research, the etiology and underlying mechanisms of periodontitis are still not fully understood. Recent studies have started to demonstrate that periodontitis is a multifactorial disease that manifests as tissue damage, and is driven by three main risk factors: bacterial, host, and environmental.^11^ In addition to smoking, which is an archetypal environmental factor,^12^ circadian disruption has also been recognized as a pivotal environmental factor that is closely related to periodontitis. Circadian disruption caused by sleep deprivation and jetlag has been reported to exacerbate periodontitis, respectively.^13,14^ It is notable that shift work, which is characterized by work schedules outside conventional daytime hours,^15^ is thought to be a significant cause of circadian disruption due to the excessive light exposure at night and delayed sleep onset.^16^ As a result of service and manufacturing demands, about 20% to 30% of the global workforce^16,17^ is engaged in this work pattern. Accumulating epidemiological evidence indicates that circadian disruption due to shift work is closely associated with an increased risk of periodontitis,^18,19^ but whether shift work-related circadian disruption functions in periodontitis remains unknown.

In the present work, we demonstrated that simulated shift work disrupted circadian rhythms and downregulated the expression of the core circadian protein BMAL1. This resulted in the increased Gasdermin D (GSDMD)-mediated pyroptosis of periodontal fibroblasts, which further contributed to the destruction of periodontal tissues and exacerbated disease progression. These findings shed light on potential strategies for developing interventions targeting the circadian clock to alleviate circadian disruption-aggravated periodontitis.

## Results

### Circadian disruption by simulated shift work aggravates periodontitis in mice

**Fig. 1:**
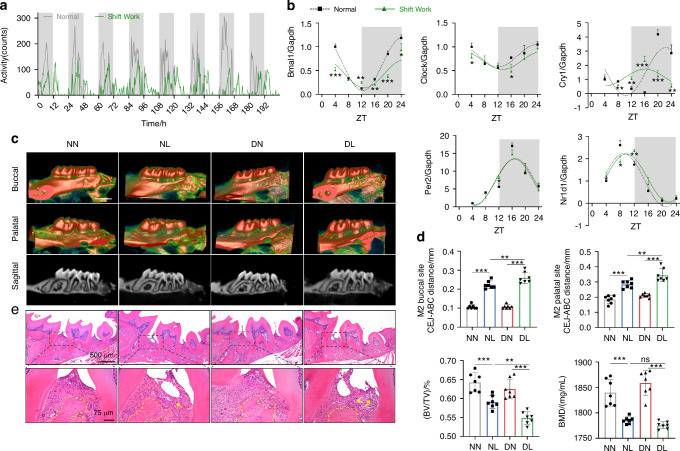
Circadian disruption by shift work simulation aggravates experimental periodontitis in mice. **a** The intensity and diurnal oscillation of physical activity in mice under normal or simulated shift work conditions measured by voluntary running wheel assay. **b** Relative mRNA expressions of indicated circadian clock genes in gingiva of mice under normal or simulated shift work conditions (*n* = 3). **c** The representative sagittal tridimensional and bidimensional views of the maxillary molars scanned by micro-CT (scale bar: 500 μm). **d** Quantification of the BMD, BV/TV, and the distance from the CEJ to the ABC in indicated groups (*n* = 7). **e** H&E staining of paraffin-embedded sections (scale bar: 500 μm; 75 μm). Data are represented as the mean ± SD. ns, no significance; *P < 0.05; **P < 0.01; ***P < 0.001. Abbreviations: ZT zeitgeber time, NN mice under normal light-dark cycles without ligature, NL mice under normal light-dark cycles with ligature, DN mice under disturbed light-dark cycles without ligature, DL mice under disturbed light-dark cycles with ligature, BMD bone mineral density, BV/TV bone volume fraction, CEJ cement-enamel junction, ABC alveolar bone crest, H&E hematoxylin and eosin

Firstly, we constructed the model of circadian disruption by controlling the light-dark cycles to simulate the condition of shift work. Voluntary running wheel assay was used to monitor the intensity and diurnal oscillation of physical activity in mice and the results showed that shift work simulation perturbed their circadian behaviors, as evidenced by a decrease in activity intensity and diurnal difference, as well as an abnormal activity phase shift (Fig. 1a). Given that circadian clock proteins act as the molecular switch controlling circadian network,^5^ we further determined the diurnal mRNA expressions of *Bmal1*, *Clock*, cryptochrome circadian regulator 1 (*Cry1*), period circadian regulator 2 (*Per2*), and nuclear receptor subfamily 1 group D member 1 (*Nr1d1*) in the gingiva of mice. Cosine curves were fitted to the individual values of mRNA expression for these genes, demonstrating the circadian variations of circadian clock genes in gingiva (Fig. 1b). And the area under the curve of *Bmal1* mRNA expression was decreased after exposure to disturbed light-dark cycles, suggesting that the transcription of *Bmal1* was downregulated by shift work simulation (Supplementary Fig. 1a). Meanwhile, under the disturbed light-dark cycle condition, the circadian amplitudes of *Bmal1* and *Cry1* mRNA expression, as well as the baseline of *Bmal1*, *Clock*, and *Cry1* mRNA expression, were reduced (Supplementary Fig. 1b, c), and there was a phase shift in *Cry1* and *Nr1d1* mRNA expression (Supplementary Fig. 1d). These results indicated a perturbed diurnal oscillation of circadian clock gene expression following exposure to the shift work simulation. Moreover, the protein levels of these genes were in chaos compared with the regular circadian oscillation (Supplementary Fig. 1e), further verifying shift work simulation resulted in the circadian rhythm disruption.

To explore the influence of circadian disruption by shift work on periodontitis progression, we constructed mouse models of circadian disruption complicated with periodontitis. The four groups were labeled as follows: NN group (normal circadian rhythm without ligature), NL group (normal circadian rhythm with ligature), DN group (circadian rhythm disruption without ligature), and DL group (circadian rhythm disruption with ligature), respectively (Supplementary Fig. 1f). Micro-computed tomography (CT) was used to evaluate the extent of alveolar bone loss, which showed that the periodontal ligature caused a decrease in alveolar bone height and an increase in root exposure. Additionally, circadian disruption by shift work accelerated these effects, as evidenced by the greater distances from the cement-enamel junction (CEJ) to the alveolar bone crest (ABC) at the buccal and palatal sites of the maxillary second molar in the DL group compared to those in the NL group. And the bone volume fraction (BV/TV) was decreased in the DL group in contrast to that in the NL group (Fig. 1c, d). Besides, hematoxylin and eosin (H&E) staining was used to assess the periodontal inflammatory condition, and the results showed significant periodontal fiber damage, augmented alveolar bone loss, and increased inflammatory cell infiltration in the DL group compared with the NL group (Fig. 1e). These results suggested that circadian rhythm disruption by shift work exacerbates experimental periodontitis.

### Progression of circadian disruption-accelerated periodontitis is associated with circadian entrainment and pyroptosis

**Fig. 2:**
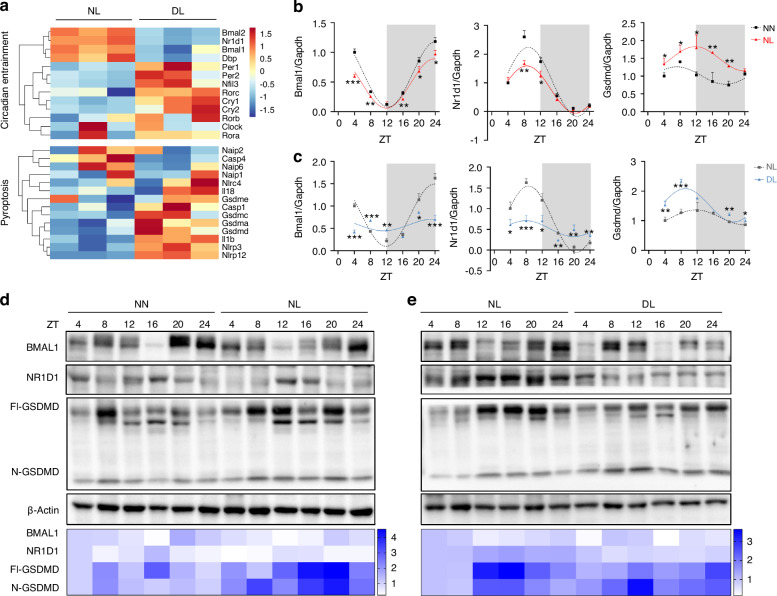
Circadian entrainment and pyroptosis are involved in the development of circadian disruption-accelerated periodontitis. **a** Heatmap of the expressions of genes related to circadian entrainment and pyroptosis (*n* = 3). **b**, **c** The diurnal rhythms of *Bmal1*, *Nr1d1*, and *Gsdmd* measured by qRT-PCR in the gingiva of mice at indicated times (*n* = 3). **d**, **e** Quantification of the diurnal oscillation of BMAL1, NR1D1, as well as Fl-GSDMD and N-GSDMD at indicated times by western blot (*n* = 3). Data are represented as the mean ± SD. *P < 0.05; **P < 0.01; ***P < 0.001. Abbreviations: NN mice under normal light-dark cycles without ligature, NL mice under normal light-dark cycles with ligature; DL mice under disturbed light-dark cycles with ligature; Fl-GSDMD full-length of GSDMD, N-GSDMD N-terminal cleavage fragment of GSDMD

Given that the mRNA expression of *Bmal1* was significantly downregulated among the detected circadian genes, and was most significantly decreased at zeitgeber time (ZT) 4 under circadian disruption, we performed a genome-wide RNA sequencing of mouse gingiva at ZT 4 to investigate the underlying mechanism by which circadian rhythm disruption accelerates periodontitis. This allowed us to obtain the transcriptional profiles of inflammatory gingiva under normal or disrupted circadian rhythm, and to delineate the cellular and molecular processes involved. In the DL group, 386 genes were found to be upregulated and 231 genes were downregulated compared to the NL group (Supplementary Fig. 2a). The KEGG enrichment results indicated that in the term of Organismal Systems, the differentially expressed genes were enriched in the environmental adaptation-related circadian entrainment process and the immune system-related NOD-like receptor (NLRs) signaling pathway (Supplementary Fig. 2b). NLRs signaling pathway plays a central role in immunity and inflammation via sensing pathogen-associated molecular patterns by NLRs.^20^ Among these NLRs, activation of NOD-like receptor protein 3 (NLRP3) results in the assembly of inflammasome and further activates GSDMD-mediated pyroptosis,^21,22^ which played an important role in inflammation.^23^ Therefore, we concentrated on genes linked to circadian entrainment and pyroptosis. We found a reduction in the mRNA expression of circadian-related genes *Bmal1* and *Nr1d1*, as well as an increase in the mRNA expression of pyroptosis-related genes *Nlrp3*, *Caspase1*, *Il-1β*, and *Gsdmd* in the DL group compared to the NL group (Fig. 2a).

We further explored the chronological expressions of central circadian genes *Bmal1* and *Nr1d1*, as well as the corresponding levels of pyroptosis effector *Gsdmd*. The circadian variations of mRNA expression for these genes during a 24-hour light-dark cycle fitted cosine curves (Fig. 2b, c). The area under the curve of *Bmal1* and *Nr1d1* mRNA expression was decreased, while that of *Gsdmd* mRNA expression was expanded in inflammatory gingiva with normal circadian rhythm (Supplementary Fig. 2c). And these phenomena were more pronounced in the inflammatory gingiva with circadian disruption (Supplementary Fig. 2d). These findings suggested that the circadian disruption-accelerated periodontitis was associated with the decline of *Bmal1* and *Nr1d1* expression and the greater activity of pyroptosis. Interestingly, we also observed an inverse trend between the protein levels of BMAL1 and GSDMD over time. Specifically, the time when GSDMD expression peaked coincided with the time when BMAL1 levels were at their trough (Fig. 2d, e), indicating BMAL1 may play a potential role in GSDMD-mediated pyroptosis regulation.

### The circadian clock protein BMAL1 functions in the regulation of GSDMD-mediated pyroptosis

To further clarify the role of BMAL1 in regulating pyroptosis in vitro, we first accessed the histological distribution of GSDMD in periodontal tissues under inflammatory conditions. We found that GSDMD was primarily distributed in epithelial and subepithelial connective tissues and was closely associated with gingival fibroblasts (Supplementary Fig. 3a, b). Thus, primary mouse gingival fibroblasts (mGFs) were obtained with tissue block enzymatic digestion (Supplementary Fig. 4a). And as a result of the cytotoxicity and BMAL1 level assay under different concentration gradients, a concentration of 5 μg/mL of *Porphyromonas gingivalis*-derived lipopolysaccharide (*P. gingivalis* LPS) was employed to treat mGFs and simulate the inflammatory challenge (Supplementary Fig. 4b, c).

**Fig. 3:**
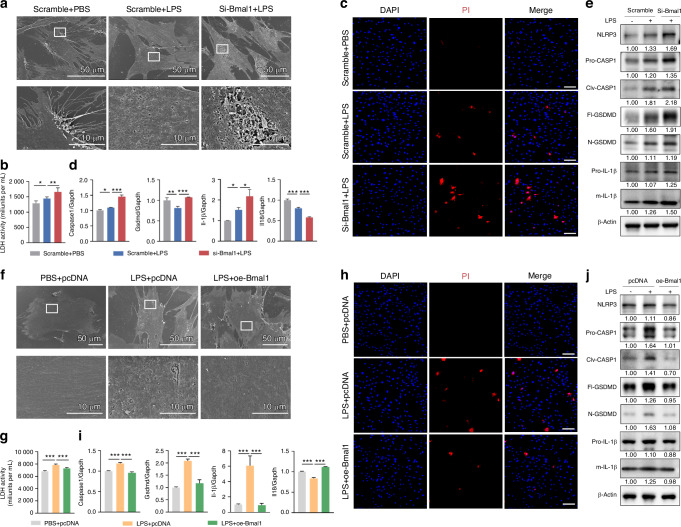
BMAL1 functions in the regulation of GSDMD-mediated pyroptosis. **a**, **f** The representative images of cell morphology in indicated groups examined by SEM (scale bar: Low magnification = 50 μm; High magnification = 10 μm) **b**, **g** Quantification of LDH activity in mGFs treated with *P. gingivalis* LPS and *Bmal1* siRNA (*n* = 5) or overexpression plasmid (*n* = 4). **c**, **h** The representative images of PI staining in indicated groups were examined by LCFM (scale bar: 100 μm). **d**, **i** Relative mRNA expressions of genes related to GSDMD-mediated pyroptosis in indicated groups measured by qRT-PCR (*n* = 3). **e**, **j** Relative levels of proteins related to NLRP3 inflammasome signaling in indicated groups detected by western blot (*n* = 3). Data are represented as the mean ± SD. *P < 0.05; **P < 0.01; ***P < 0.001. Abbreviations: LPS lipopolysaccharide, LDH lactate dehydrogenase, PI propidium iodide, SEM scanning electron microscope, LCFM laser confocal fluorescence microscopy

To explore the effect of BMAL1-downregulation on GSDMD-mediated pyroptosis, *Bmal1* siRNA was applied to mGFs prior to *P. gingivalis* LPS (Supplementary Fig. 5a). The qRT-PCR assay demonstrated that the transfection of *Bmal1* siRNA suppressed its transcription (Supplementary Fig. 5b). And that worsened the cell membrane perforation, cell rupture, and cell death, which were caused by *P. gingivalis* LPS, as respectively confirmed by scanning electron microscope (SEM) detection (Fig. 3a), lactate dehydrogenase (LDH) activity assay (Fig. 3b), and propidium iodide (PI) staining (Fig. 3c). Meanwhile, the mRNA expressions of pyroptosis-related genes, including *Caspase1*, *Gsdmd*, and *Il-1β*, were found to be upregulated following *Bmal1* interference in comparison to sole LPS treatment (Fig. 3d). The western blot assay demonstrated that *Bmal1* interference further increased the level of *P. gingivalis* LPS-induced NLRP3 and the cleavages of CASPASE1, GSDMD, and IL-1β (Fig. 3e). Additionally, the *Bmal1* overexpression plasmid was transfected in *P. gingivalis* LPS-treated mGFs to clarify the protective effect of BMAL1 on pyroptosis in an inflammatory environment (Supplementary Fig. 5d). The overexpression efficiency was verified by qRT-PCR (Supplementary Fig. 5e). The results of SEM detection, LDH activity assay, and PI staining showed that overexpression of *Bmal1* alleviated the cell membrane perforation (Fig. 3f), cell rupture (Fig. 3g), and cell death (Fig. 3h) caused by *P. gingivalis* LPS treatment. Besides, the mRNA expressions of *Caspase1*, *Gsdmd*, and *Il-1β* (Fig. 3i) as well as the protein level of NLRP3 and the cleavages of CASPASE1, GSDMD, and IL-1β (Fig. 3j) were further reduced after *Bmal1* overexpression compared to sole LPS treatment. And the results of GSDMD immunofluorescence were also consistent with the above results (Supplementary Fig. 5c, f). These data illustrated that BMAL1 can repress GSDMD-mediated pyroptosis via regulating NLRP3 inflammasome signaling.

### BMAL1 regulates *Gsdmd* transcription independent of NR1D1

**Fig. 4:**
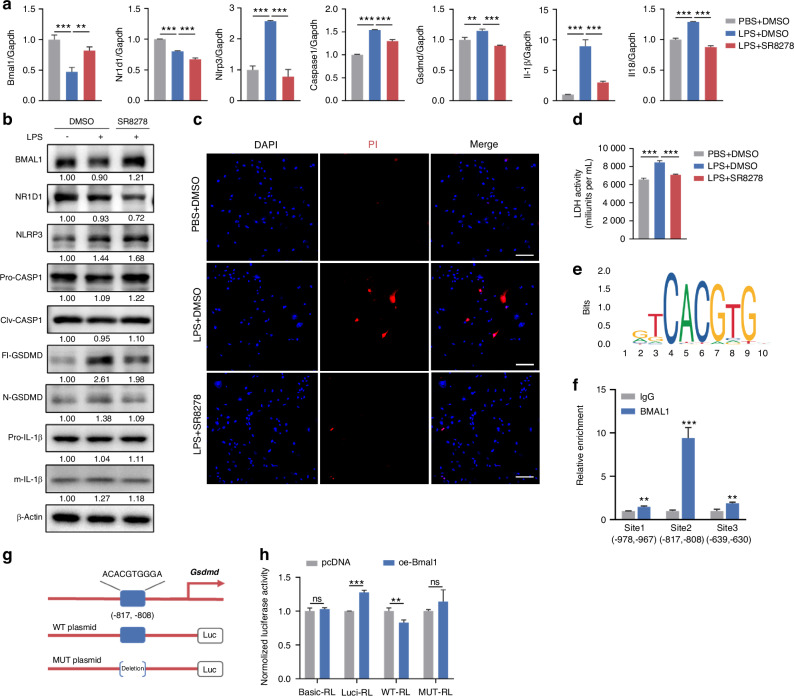
BMAL1 regulates *Gsdmd* transcription independent of NR1D1. **a** Relative mRNA expressions of *Bmal1*, *Nr1d1*, and genes related to NLRP3 inflammasome signaling in indicated groups measured by qRT-PCR (*n* = 3). **b** Relative expressions of BMAL1, NR1D1, and proteins related to NLRP3 inflammasome signaling in indicated groups detected by western blot (*n* = 3). **c** The representative images of PI staining in indicated groups examined by LCFM (scale bar: 100 μm). **d** Quantification of LDH activity in indicated groups (*n* = 4). **e** Sequence logos of consensus DNA binding sites for genes regulated by BMAL1. **f** Verification of the binding between BMAL1 and the *Gsdmd* promoter region by ChIP assay. **g** The binding sites of BMAL1 and *Gsdmd*, and the design for luciferase reporters. **h** Normalized luciferase activity after co-transfection of pcDNA or *Bmal1* plasmid together with GSDMD-WT or GSDMD-MUT luciferase reporters measured by dual luciferase assay (*n* = 3). Data are represented as the mean ± SD. ns, no significance; **P < 0.01; ***P < 0.001. Abbreviations: LPS lipopolysaccharide, LCFM laser confocal fluorescence microscopy, PI propidium iodide, LDH lactate dehydrogenase; WT wild type, MUT mutant type

BMAL1 upregulates the transcription of *Nr1d1*, and it has been evidenced that BMAL1 and NR1D1 get involved in inhibiting NLRP3 inflammasome pathway, respectively.^24,25^ However, it was unclear whether BMAL1 regulated GSDMD-mediated pyroptosis through modulating NR1D1. Therefore, we used SR8278, the specific antagonist of NR1D1,^26^ which is also an agonist of BMAL1,^27^ for the *P. gingivalis* LPS-treated mGFs (Supplementary Fig. 6a). Although the mRNA expressions of *Nlrp3*, *Caspase1*, *Gsdmd*, *Il-1β*, and *Il18* were decreased following SR8278 treatment (Fig. 4a), the protein levels of BMAL1, NLRP3, and pro-CASPASE1 were increased. Interestingly, the full length and N-terminal cleavage of GSDMD were downregulated in mGFs treated with SR8278 compared to sole LPS treatment (Fig. 4b). Meanwhile, the immunofluorescence results indicated that SR8278 decreased the elevated total protein level of GSDMD induced by *P. gingivalis* LPS (Supplementary Fig. 6b). Notably, SR8278 ameliorated *P. gingivalis* LPS-induced cell death (Fig. 4c) and cell rupture (Fig. 4d). These results suggested that BMAL1 repressed NLRP3 inflammasome signaling through regulating NR1D1, and other ways may exist for BMAL1 to modulate the pyroptosis effector GSDMD. As BMAL1 has been evidenced to bind to genes containing E-box motifs (Fig. 4e) and regulate their transcription,^28^ we further predicted the binding sites of BMAL1 to the *Gsdmd* promoter using JASPAR (https://jaspar.elixir.no/). And the ChIP assay confirmed the direct binding of BMAL1 to the *Gsdmd* promoter region (Fig. 4f). To further verify the effect of BMAL1 on *Gsdmd* transcription, plasmids containing the motif “ACACGTGGGA” or not were constructed (Fig. 4g), and a dual-luciferase reporter assay was performed, which showed that *Bmal1* overexpression significantly decreased the luciferase activity of the promoter construct containing the above motif (Fig. 4h). These findings indicated the repressive effect of BMAL1 on *Gsdmd* transcription.

### GSDMD-mediated pyroptosis aggravates periodontitis progression

**Fig. 5:**
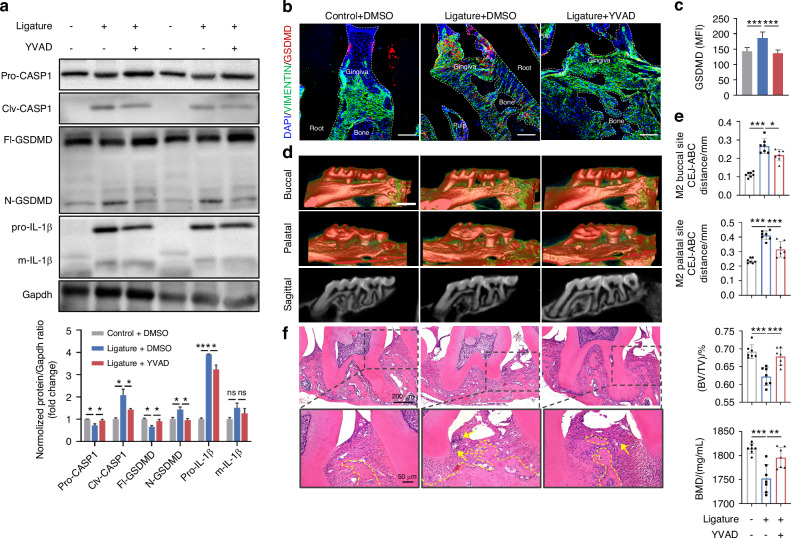
Inhibition of GSDMD-mediated pyroptosis improves the progression of periodontitis. **a** Relative expressions of proteins related to GSDMD-mediated pyroptosis in the gingiva of mice with periodontal ligature and periodontal local injection of YVAD. **b, c** Representative images (scale bar: 100 μm) and quantitative analysis of GSDMD expression in indicated groups by immunofluorescence staining (*n* = 6). **d** The representative sagittal tridimensional and bidimensional views of the maxillary molars in indicated groups scanned by micro-CT (scale bar: 500 μm). **e** Quantification of the distance from CEJ to the ABC as well as the index of BV/TV and BMD in indicated groups (*n* = 7). **f** Representative images of H&E staining of the gingiva in indicated groups (scale bar: 200 μm; 50 μm). Data are represented as the mean ± SD. ns, no significance; *P < 0.05; **P < 0.01; ***P < 0.001. Abbreviations: BMD bone mineral density, BV/TV bone volume fraction, CEJ cement-enamel junction, ABC alveolar bone crest, H&E hematoxylin and eosin

The above results indicated that the BMAL1-GSDMD axis might be a potential target for regulating cell rupture and cell death, thereby affecting the progression of periodontitis under circadian disruption. To verify this hypothesis, we first clarified the role of GSDMD-mediated pyroptosis acted in periodontitis progression. As the results of immunohistochemistry showed, the pyroptosis-related protein levels of N-GSDMD, IL-1β, and IL-18, as well as the inflammatory factor TNF-α were significantly augmented in human periodontitis tissues compared with those in the healthy tissues (Supplementary Fig. 7). Therefore, we constructed the mice periodontitis model combined with periodontal injection of the pyroptosis inhibitor, YVAD.^29^ The western blot results showed that periodontal ligature increased the protein level of pro-IL-1β and enhanced the cleavages of CASPASE1 and GSDMD, while YVAD injection mitigated these effects (Fig. 5a). Besides, results from immunofluorescence further confirmed that YVAD ameliorated the upregulated GSDMD levels in gingival fibroblasts induced by periodontal ligature (Fig. 5b, c). Additionally, micro-CT and H&E staining showed that with the inhibition of GSDMD-mediated pyroptosis, alveolar bone resorption, periodontal inflammation, and fiber destruction (Fig. 5d–f) due to periodontal ligature were also significantly improved.

### BMAL1-upregulation alleviates circadian disruption-accelerated periodontitis via lessening GSDMD-mediated pyroptosis

**Fig. 6:**
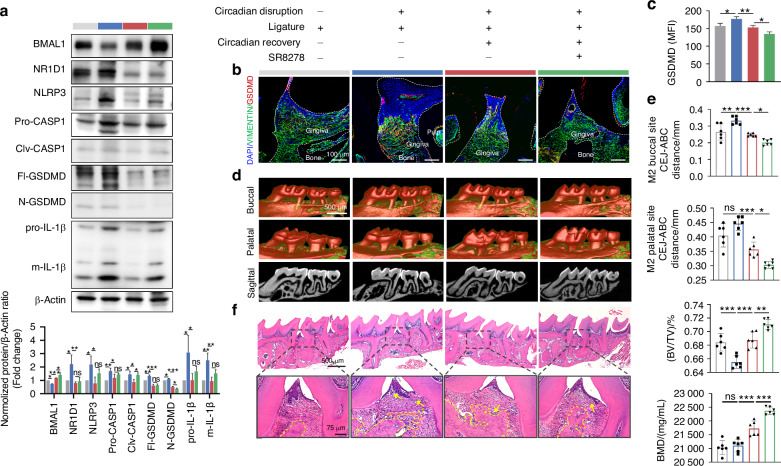
BMAL1-upregulation alleviates circadian disruption-accelerated periodontitis via lessening GSDMD-mediated pyroptosis. **a** Representative images and quantitative analysis of BMAL1, NR1D1, and proteins related to NLRP3 inflammasome signaling in indicated groups detected by western blot (*n* = 3). **b**, **c** Representative images (scale bar: 100 μm) and quantification of GSDMD expression in indicated groups by immunofluorescence staining (*n* = 3). **d** The representative sagittal tridimensional and bidimensional views of the maxillary molars in indicated groups scanned by micro-CT (scale bar: 500 μm). **e** Quantification of the BMD, the BV/TV, and the distance from the CEJ to the ABC in indicated groups (*n* = 6). **f** Representative images of H&E staining of the gingiva in indicated groups (scale bar: 500 μm; 75 μm). Data are represented as the mean ± SD. ns, no significance; *P < 0.05; **P < 0.01; ***P < 0.001. Abbreviations: BMD bone mineral density, BV/TV bone volume fraction, CEJ cement-enamel junction, ABC alveolar bone crest, H&E hematoxylin and eosin

Since BMAL1 restrained GSDMD-mediated pyroptosis that accelerated periodontitis progression, and BMAL1 was repressed in the gingiva of circadian disruption-related periodontitis, we hypothesized that recovering the level of BMAL1 could be a feasible approach to alleviating circadian disruption-aggravated periodontitis. Accordingly, after constructing the model of circadian disruption complicated with periodontitis, we removed the ligatures, recovered the circadian rhythm to normal condition, and administered SR8278 intraperitoneally every 2 days (Supplementary Fig. 8a). Since at ZT 4, the mRNA expression of *Bmal1* under normal circadian rhythm and its decline under circadian disruption were second only to ZT 24, and it was a time point that was more convenient for experimental operation compared to ZT 24, we selected ZT 4 for SR8278 administration. As the results of immunofluorescence showed, circadian rhythm restoration corrected the reduction of BMAL1 caused by circadian disruption, and injection of SR8278 at ZT 4 further improved that (Supplementary Fig. 8b, c). The results from western blot of gingiva showed that circadian recovery improved the decreased BMAL1 and the increased levels of NLRP3, CASPASE1, GSDMD, and IL-1β, as well as the elevated cleavages of CASPASE1, GSDMD, and IL-1β, which were caused by circadian disruption. After SR8278 injection, the level of BMAL1 was further promoted. Although the protein levels of NLRP3, CASPASE1, and IL-1β, as well as the cleavages of CASPASE1 and IL-1β, showed an upregulated trend, the N-terminal cleavages of GSDMD were significantly downregulated following SR8278 injection (Fig. 6a). Consistently, the immunofluorescence results showed that circadian recovery reversed the increased total protein level of GSDMD caused by circadian disruption, and SR8278 injection further alleviated that (Fig. 6b, c). Subsequently, micro-CT and H&E staining were performed to estimate the alveolar bone resorption and the inflammatory condition of periodontal tissues. And the results showed that the alveolar bone resorption and periodontal destruction were aggravated when GSDMD were upregulated caused by circadian disruption, and were alleviated with the reduced GSDMD level due to circadian recovery and SR8278 injection (Fig. 6d–f). Taken together, these results indicated that restoring the level of BMAL1 alleviates circadian disruption-aggravated periodontitis via lessening GSDMD-mediated pyroptosis.

## Discussion

**Fig. 7:**
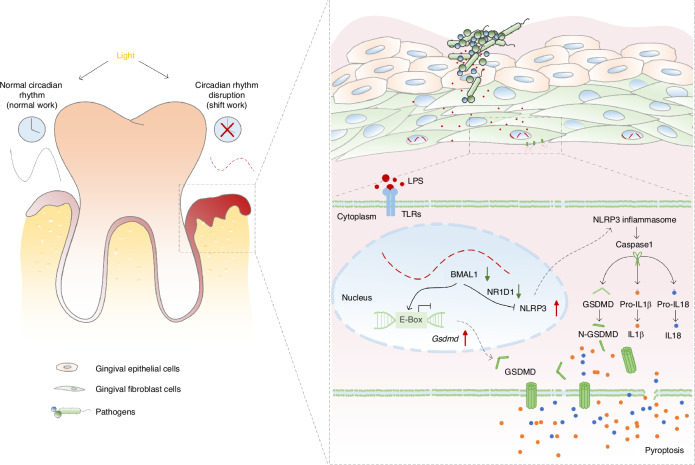
Circadian disruption caused by shift work simulation aggravates periodontitis via orchestrating GSDMD-mediated pyroptosis through BMAL1 in gingival fibroblasts

Circadian disruption has been implicated in the progression of periodontitis,^13,14^ but the effects of circadian disruption caused by shift work on periodontitis and its underlying mechanisms remain unclear. In this study, we revealed that circadian disruption by shift work accelerates periodontitis progression with impairing the time-of-day variations of circadian clock proteins and pyroptosis-related proteins in gingiva. Mechanistically, the circadian transcription factor BMAL1 negatively regulates the NLRP3 inflammasome signaling pathway that provokes GSDMD-mediated pyroptosis through modulating NR1D1, and also directly affects *Gsdmd* transcription via controlling the E-box elements in the *Gsdmd* gene promoter. The periodic BMAL1 expression is perturbed when the circadian rhythm is disrupted, resulting in the dysregulation of fibroblasts pyroptosis and further exacerbating periodontitis progression (Fig. 7).

Rhythmic physiological functions that change with the circadian rhythm synchronized by light-dark cycles play an important role in the homeostasis maintenance.^4^ However, circadian rhythm disruption occurs when the internal circadian periods are out of sync with the environmental light-dark cycles due to various factors like constant light exposure, shift work, and other lifestyle changes. This phenomenon is becoming increasingly prevalent in modern society and has been linked to various diseases.^5^ Previous epidemiological studies have shown that circadian disruption induced by shift work is closely associated with an increased risk for periodontal disease,^18,19^ while the mechanisms remain unclear. Prior experimental studies have verified that sleep deprivation exacerbates periodontitis progression, accompanied with a downregulated BMAL1 and an upregulated oxidative stress and apoptosis.^13^ Additionally, circadian disruption caused by jetlag affects periodontitis by modulating macrophage activity and alveolar bone homeostasis.^14^ Consistently, our finding demonstrated that the circadian disruption by disturbed light-dark cycles, which aims to simulate shift work conditions, accelerates periodontal tissue destruction.

We further found that this process is closely related to circadian entrainment, which is defined as the entrainment of the endogenous clock by recurring exogenous signals to synchronize the physiological rhythms of organism to environmental cues.^30^ Besides, this process is also associated with pyroptosis, which is an inflammatory programmed cell death caused by the N-terminal domain of GSDMD or other Gasdermin family proteins, resulting in the perforation of cell membrane, the release of cytosolic contents, and a widespread and intense inflammatory response.^31^ According to the data of mRNA sequencing, we detected diurnal variations of the central clock proteins BMAL1 and NR1D1, as well as the pyroptosis executioner GSDMD. Interestingly, the total protein levels of BMAL1 and NR1D1 are reduced in inflammatory gingiva, and are further downregulated under circadian disruption conditions, whereas GSDMD shows an opposite trend. Consistent with our findings, it has been reported that the mRNA expressions of BMAL1 and NR1D1 are reduced in inflammatory aortas due to NF-κB signaling activated by *P. gingivalis*, which represses the expression of BMAL1 through facilitating heterodimer p65/p50 nuclear translocation and recruiting DNMT-1 to promote *Bmal1* methylation.^32^ Moreover, the declines of NR1D1 have been also observed in inflammatory colons and in the colons of jetlag mice.^33^ Besides, *Bmal1* mRNA is suppressed in skins^24^ and distal femoral growth plates^34^ of jetlag mice. Notably, BMAL1 exerts an important influence on inflammation regulation. BMAL1-downregulation aggravates atherosclerosis by affecting oxidative stress,^32^ and mice harboring *Bmal1*-deficient regulatory T cells exhibits increased visceral adipose tissue inflammation.^35^ Plus, our data revealed that the protein levels of BMAL1 and GSDMD display a reverse trend over time, we therefore speculated that the decreased BMAL1 is involved in the regulation of GSDMD-mediated pyroptosis, contributing to periodontitis progression.

Fibroblasts have recently been evidenced to function in inflammation regulation as key sentinel immune cells.^36,37^ The extent of fibroblasts pyroptosis is in line with the progression of apical periodontitis.^38^ Our data showed the histological distribution of GSDMD is closely related to fibroblasts. Thus, we further validated the regulatory roles of BMAL1 in pyroptosis by using fibroblasts and found that variations in GSDMD-mediated pyroptosis are inversely correlated with interference or overexpression of BMAL1. This is attributed to its modulation role in the NLRP3 inflammasome pathway. A prior study also reported that overexpressed BMAL1 in macrophages protected against pyroptosis by inhibiting NLRP3 inflammasome signaling.^39^ Meanwhile, *Nr1d1*, as the target gene of BMAL1, can suppress the transcription of *Nlrp3* and *Il-1β*, thereby controlling the activation of NLRP3 inflammasome pathway.^33,40^ However, *Bmal1* overexpression failed to modulate *Propionibacterium acnes*-induced pro-inflammatory factors CXCL1, IL-1α, and IL-6 in *Nr1d1*-silenced mouse keratinocytes.^24^ Thus, we assessed whether the impact of BMAL1 on the NLRP3 inflammasome pathway is dependent on NR1D1 by using SR8278, the antagonist of NR1D1^26^ as well as the agonist of BMAL1.^27^ Interestingly, although SR8278 promotes the protein levels of NLRP3 and CASPASE1, which is consistent with the previous studies,^25^ the cell rupture and death are significantly improved with the decreased expressions and cleavages of GSDMD. This suggests that GSDMD is the key to pyroptosis, and other approach exists in the regulation of GSDMD by BMAL1, independent of NR1D1. Given that BMAL1 as a transcription factor, can control the mRNA expression of target genes via direct binding to the E-box elements of their promoters,^41^ we further validated BMAL1 is a direct negative regulator of *Gsdmd* transcription and activity. Our findings reveal a novel aspect of BMAL1 function in modulating pyroptosis.

Pyroptosis presents in variety types of periodontal cells, contributing to the activation and recruitment of immune cells to protect against pathogens. However, the continuous increase in cell death and release of inflammatory factors caused by pyroptosis may lead to periodontitis progression.^42^ Thus, we demonstrated that GSDMD-mediated pyroptosis performs an important role in periodontitis modulation, as evidenced by the fact that inhibiting pyroptosis significantly improves periodontal destruction. Furthermore, restoring normal circadian rhythm and using SR8278 activates the expressions of BMAL1 and inhibits GSDMD-mediated pyroptosis, thereby ameliorating circadian disruption-accelerated periodontitis progression. In line with our findings, restoring normal levels of BMAL1 by melatonin inhibits pyroptosis by reducing NF-κB signaling that positively regulated the transcription of GSDMD.^43^

Although our study provides new insights into the mechanisms of circadian disruption-deteriorated periodontitis and the previously undiscovered roles of BMAL1 in GSDMD-mediated pyroptosis, some limitations should be noted. First, we constructed the circadian disruption model based on shift work, but the patterns of shift work are random and vary among the population.^17^ Therefore, the experimental results can only represent the changes under the specific modeling condition. A recent study reported a model of shift work different from this study, which triggered circadian rhythm disruption and exacerbated reperfusion injury in myocardial infarction.^44^ This indicates that our conclusions may have universal applicability, but further model experiments are necessary to confirm this. Second, further examinations are needed to investigate whether BMAL1 regulates GSDMD-mediated pyroptosis in ways other than the transcriptional regulation of *Gsdmd*. For instance, whether BMAL1 is involved in modulating the cleavage of GSDMD or regulating pyroptosis though membrane repair.^45^ Moreover, in addition to pyroptosis, BMAL1 contributes to regulating many other processes, including apoptosis^46^ and oxidative stress,^41,47^ whose dysregulation also affects inflammation progression. Their roles in circadian disruption-related periodontitis still await further exploration.

In conclusion, we illustrated that circadian rhythm disruption by simulated shift work exacerbates the progression of experimental periodontitis via activating fibroblast pyroptosis in gingiva. Moreover, we revealed that BMAL1/GSDMD signaling acts as a coordinator of shift work-related circadian disruption and periodontitis progression, providing a potential guidance for the development of intervention strategies targeting circadian clock against periodontitis.

## Materials and methods

### Mice

Six-week-old C57BL/6J male mice were kept under normal light-dark cycles, with lights turned on at 6 A.M. and turned off at 6 P.M. To induce circadian rhythm disruption by simulated shift work, the mice were exposed to a condition where the lights were turned on 8 h in advance every 1–2 days for 8 weeks, i.e., turning the lights on at 10 P.M. and off at 10 A.M. according to the previous methods.^48,49^ For voluntary running wheel assessing, mice were placed in individual cages equipped with voluntary running wheels and exposed to normal or disturbed light-dark cycles for 8 days. Activity profiles were generated by calculating the counts of running wheel spins per 30 min. To construct the model of circadian rhythm disruption complicated with periodontitis, wire was ligated around the first and second maxillary molars of mice for 14 days in the seventh week of abnormal light exposure. To inhibit pyroptosis, Z-YVAD-FMK (MedChem Express, HY-P1009, Monmouth Junction, NJ, USA) was administered into three palatal gingival sites of the experimental maxillary molars every 2 days at a concentration of 200 μmol/L (2 μL) per site using a 33-gauge needle Hamilton syringe (Hamilton Company, Reno, NV, USA) at ZT 4 (10 A.M.). For restoring the level of BMAL1, SR8278 (MedChem Express, HY-14415) was injected intraperitoneally at a concentration of 500 μmol/L (110 μL) at ZT 4. All animal studies were carried out in accordance with the committee guidelines of the Animal Care Committee of the Fourth Military Medical University (Approval No. IACUC-20230117).

### Micro-CT

The maxillae were collected and fixed in 4% paraformaldehyde for 24 h and then scanned using the Quantum GX2 Micro-CT Imaging System (PerkinElmer, Waltham, MA, USA). The indicators including the bone mineral density (BMD), BV/TV, and the distance from the CEJ to the ABC were used to evaluate the degree of alveolar bone loss and tissue damage.

### RNA extraction and quantitative real-time PCR (qRT-PCR)

For tissue RNA extraction, the buccal and palatal gingiva of the experimental second maxillary molars were collected. Cell samples were harvested as usual. After being washed three times with phosphate-buffered saline (PBS; Procell Life Science & Technology, Wuhan, China), the gingiva or cells were immersed in the AG RNAex Pro Reagent (Accurate Biology, AG21102, Changsha, China). Total RNA was extracted following the manufacturer’s instructions. An Evo M-MLV RT Premix for qPCR kit (Accurate Biology, AG11706) was used to prepare the cDNA synthesis reactions according to the manufacturer’s instructions. Glyceraldehyde-3-phosphate dehydrogenase (*Gapdh*) was used as the reference gene. qRT-PCR was performed on the CFX96 Touch Real-Time PCR Detection System (Bio Rad, CA, USA) by using a SYBR® Green Premix Pro Taq HS qPCR kit (Accurate Biology, AG11701), and the relative expressions of target mRNAs were calculated by the 2^−ΔΔCT^ method. The primer sequences used in this study are listed in Table S1.

### Western blot

The proteins were harvested from gingiva and cells by using Cell Lysis Buffer for Western and IP (Beyotime, P0013J, Shanghai, China) with ProtLytic Protease Inhibitor Cocktail (New Cell & Molecular Biotech, P001, Suzhou, China). After separated by Tris-glycine SDS-polyacrylamide gel electrophoresis (7.5% or 10%), the proteins were transferred to polyvinylidene difluoride (PVDF) membranes (Merck Millipore, IPVH00010, Billerica, MA, USA) and blocked with QuickBlock™ Blocking Buffer for Western Blot (Beyotime, P0252). The PVDF membranes were incubated overnight at 4 °C with antibodies against BMAL1 (ABclonal, A17334, Wuhan, China), CLOCK (ABclonal, A7265), CRY1 (ABclonal, A6890), PER2 (ABclonal, A13168), NR1D1 (ABclonal, A20452), NLRP3 (R&D Systems, MAB7578, Minneapolis, MN, USA), CASPASE1 (Proteintech, 22915-1-AP, Wuhan, China), GSDMD (Santa Cruz Biotechnology, sc-393581, Santa Cruz, CA, USA), IL-1β (ImmunoWay Biotechnology, YT5201, Plano, TX, USA), β-Actin (ImmunoWay Biotechnology, YM3029). After incubation with Peroxidase AffiniPure Goat Anti-Mouse IgG (H + L) (Jackson, 115-035-003, West Grove, PA, USA), HRP Goat Anti-Rabbit IgG (H + L) (ImmunoWay Biotechnology, RS0002), HRP Goat Anti-Rat IgG (H + L) (ABclonal, AS028), the bands were visualized by using an enhanced chemiluminescence kit (AccuRef Scientific, AP0082S, China) and imaged by the ChemiDoc XRS+ System (Bio Rad).

### Chromatin immunoprecipitation (ChIP)

The ChIP assay was performed by using NIH3T3 cell line, which was purchased from the Cell Repository of the Chinese Academy of Sciences (Shanghai, China), according to the manufacturer’s instructions of ChIP Assay Kit (Beyotime, P2078). Briefly, upon reaching 90% confluence in a 100 mm dish, cells were cross-linked for 10 min using 1% formaldehyde (Sigma-Aldrich, F8775, MO, USA). Genomic DNA was fragmented to a size of 200 to 500 bp using sonication, and immunoprecipitated with either an anti-BMAL1 antibody (Cell Signaling Technology, 14020S, MA, USA) or an IgG antibody (Proteintech, 30000-0-AP). The genomic DNA that had been detached and purified was amplified by qRT-PCR with the primers listed in Table S2.

### Inclusion and exclusion criteria of clinical periodontal tissue collection

The collection of clinical samples has been approved by the Ethics Committee of the Stomatology Hospital of the Fourth Military Medical University (Approval No. IRB-REV-2022046). The healthy periodontal tissues involved in the experiment were collected from the healthy third molars or premolars, which were extracted from patients aged 18 to 35 years old, with no periodontal attachment loss, redness, and swelling caused by periodontitis. The inflammatory periodontal tissues were obtained from patients aged 18 to 35 years old who underwent flap surgery due to severe periodontitis. The clinical attachment loss of the tooth was ≥5 mm, and the X-ray showed that the alveolar bone resorption exceeded 1/2 to 2/3 of the root length, which met the diagnostic criteria for stage III to VI periodontitis. The patients of tissue origin have no systemic disease.

### Histological analysis

The gingival tissues from patients and the maxillae from mice were fixed in 4% paraformaldehyde for 24 h, after which the samples were washed with PBS several times. With or without decalcified with 10% ethylenediaminetetraacetic acid for 20 days, the samples were dehydrated using either alcohol or sucrose solution for paraffin-embedded sections or cryosections, respectively. Paraffin-embedded sections were stained with standard H&E for physiopathological analysis and immunolabeled with antibodies targeted N-GSDMD (1:500, ABclonal, A22523), IL-1β (1:200, ImmunoWay Biotechnology, YT5201), IL18 (1:100, Abcam, ab243091, Cambridge, MA, USA), TNF-α (1:100, ImmunoWay Biotechnology, YT4689) for immunohistochemistry analysis. For dual-color immunofluorescence analysis, anti-VIMENTIN (1:100, ABclonal, A19607) and anti-GSDMD (1:100) were used to identify pyroptosis in the gingiva. Images were collected by Lecia Microsystems (M205FA, Leica, Germany) and Olympus laser confocal fluorescence microscopy (LCFM, FV1000, Olympus, Japan).

### mRNA sequencing

The gingival total RNA was extracted using Trizol reagent (Thermo Fisher Scientific, 15596018, Waltham, MA, USA) following the manufacturer’s instructions. After purification, the mRNA was fragmented into short fragments and reverse-transcribed to cDNAs. Following adding an A-base, the fragmented cDNAs were ligated to the adapter containing a T-base overhang, and size selection was performed using AMPureXP beads. Amplified with PCR, the average insert size of the final cDNA libraries was (300 ± 50) bp. And at last, the 2 × 150 bp paired-end sequencing (PE150) was performed on an Illumina Novaseq™ 6000 (LC-Bio Technology, Hangzhou, China) following the vendor’s recommended protocol. Bioinformatic analysis was performed using the OmicStudio tools available at https://www.omicstudio.cn/tool.

### Cells

Primary mGFs isolated from gingival tissues of the healthy mice, were cultured in Dulbecco’s Modified Eagle’s Medium (DMEM; Gibco, Grand Island, NY, USA) supplemented with 10% fetal bovine serum (FBS; Procell Life Science & Technology), 100 U/mL penicillin, and 100 mg/mL streptomycin (BioCytoSci, Burlington, VT, USA) in a 37 °C incubator with 5% CO_2_ and 95% humidity. To simulate the inflammatory challenge, mGFs were treated with *P. gingivalis* LPS (InvivoGen, tlrl-pglps, San Diego, CA, USA) at a concentration of 5 μg/mL. For the transfection assay, when the mGFs reached 70% confluence in six-well plates, 75 pmol of *Bmal1* siRNA (Santa Cruz Biotechnology, sc-38166) or 1 μg of *Bmal1* plasmid (Gene Pharma, Shanghai, China) were transfected into mGFs using X-treme GENE HP DNA Transfection Reagent (Roche Diagnostics, 51572200, Mannheim, Germany), prior or posterior to LPS treatment. For NR1D1 inhibition, mGFs were treated with SR8278 at a concentration of 2.5 μmol/L after *P. gingivalis* LPS treatment.

### Cytotoxicity assay

Cellular cytotoxicity was detected by PI staining and LDH activity assay. Following LPS treatment and transfection, mGFs were harvested, rinsed with PBS, and stained with 10 μg/mL of PI (Beyotime, ST511) for 20 min. Subsequently, Antifade Mounting Medium containing Hoechst 33342 (Beyotime, P0133) was used for nuclear counterstaining. Images were collected by Olympus LCFM. Meanwhile, the supernatants of mGFs were centrifuged and transferred to fresh EP tubes to measure the LDH activity using CheKine™ Micro LDH Assay Kit (Abbkine Scientific, KTB1110, Atlanta, GA, USA) according to the manufacturer’s instructions. The absorbance was detected at 450 nm with a microplate spectrophotometer (BioTek, Epoch, Winooski, VT, USA).

### SEM detection

The mGFs were rinsed with PBS five times and fixed in 2.5% glutaraldehyde (Leagene, DF0156, Beijing, China) at 4 °C overnight, after which the cells were washed in PBS several times and dehydrated with graded ethanol. Following critical point drying by hexamethyldisilazane, the samples were sputtered with gold and observed using an FE-SEM (Hitachi S-4800, Japan).

### Dual-luciferase reporter assay

Wild-type (WT) mouse *Gsdmd* promoter and its mutant (MUT) deleting the E-box motif (5’-ACACGTGGGA-3’) were cloned into a GPL4-Basic luciferase reporter construct (Gene Pharma), respectively. L929 cells were transfected with the GPL4-Basic plasmid (empty vector), GPL4-Luci plasmid (control vector expressing firefly luciferase), the indicated reporter plasmids, GPL4-RL plasmid (control vector expressing Renilla luciferase), and either Bmal1 plasmid or empty vector using Lipofectamine 3000 (Thermo Fisher Scientific, Waltham, MA). After transfection for 48 h, cells were harvested and the luciferase activities were measured using a Dual-Luciferase Reporter Gene Assay Kit (Beyotime, RG027) by using a VICTOR Nivo Multimode plate reader (PerkinElmer, HH35000500, Finland).

### Statistical analysis

The data are displayed as the mean ± SD of at least three independent experiments. Data analysis was performed using GraphPad Prism 8 software (GraphPad Software, San Diego, CA, USA). Unpaired Student’s *t-*tests were used to assess the significant differences between two independent groups, while one-way or two-way ANOVA multiple comparisons were used to compare multiple independent groups. Differences were considered significant at the values of *P < 0.05, **P < 0.01, and ***P < 0.001.

## Supplementary information

Supplementary Materials

## Data Availability

The data acquired and/or analyzed during the present study are available from the corresponding author on reasonable requests.
