# Supplementary material for: Circadian disruption by simulated shift work aggravates periodontitis via orchestrating BMAL1 and GSDMD-mediated pyroptosis
Source: Int J Oral Sci. 2025 Feb 25;17:14. doi: 10.1038/s41368-024-00331-x (PMC11861291; doi:10.1038/s41368-024-00331-x)
Supplement: Supplementary file 1 — Supplementary Materials [file 41368_2024_331_MOESM1_ESM.docx]

Supplementary Materials

**Circadian Disruption by** **Simulated Shift Work Aggravates Periodontitis via** **Orchestrating** **BMAL1 and GSDMD-Mediated Pyroptosis**

Yazheng Wang^1,2^ *****, Rui Li^1,^ *****, Qingyuan Ye^2,^ ***,** Dongdong Fei^3^, Xige Zhang^1^, Junling Huang^1^, Tingjie Liu^1^, Jinjin Wang^1,^ **^†^**, Qintao Wang^1,^ **^†^**

1. State Key Laboratory of Oral & Maxillofacial Reconstruction and Regeneration, National Clinical Research Center for Oral Diseases, Shaanxi International Joint Research Center for Oral Diseases, Department of Periodontology, School of Stomatology, the Fourth Military Medical University, Xi’an 710032, People’s Republic of China

2. Key laboratory of Shaanxi Province for Craniofacial Precision Medicine Research, Department of Periodontology, College of Stomatology, Xi’an Jiaotong University, Xi’an 710004, People’s Republic of China

3. State Key Laboratory of Oral & Maxillofacial Reconstruction and Regeneration, National Clinical Research Center for Oral Diseases, Shaanxi Key Laboratory of Stomatology, Digital Dentistry Center, School of Stomatology, the Fourth Military Medical University, Xi’an 710032, People’s Republic of China

4. Department of Stomatology, the Seventh Medical Center of PLA General Hospital, Beijing 100700, People’s Republic of China

* Authors contributed equally to this work.


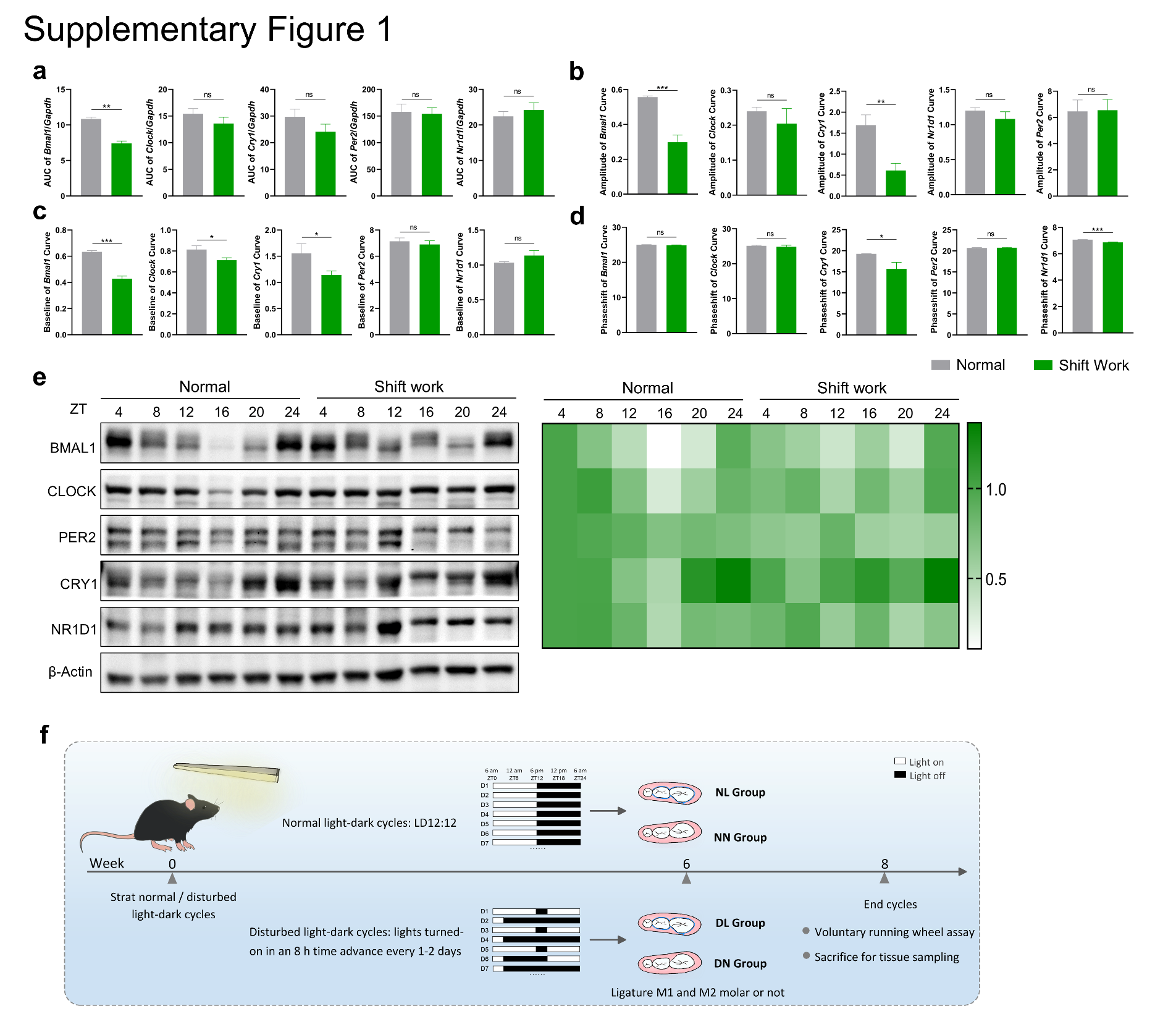


**Supplementary Figure 1.** Construction and verification of the circadian disruption model. Quantification of the area under the cosine curves **(a)** as well as the amplitude **(b)**, the baseline **(c)**, and the phase shift **(d)** of the cosine curves fitted to the diurnal mRNA expressions for *Bmal1*, *Clock*, *Cry1*, *Per2*, and *Nr1d1* in mouse gingiva under normal or simulated shift work conditions. **(e)** Expressions of indicated circadian clock proteins in mouse gingiva under normal or simulated shift work conditions (*n*=3). **(f)** Experimental design for model construction of circadian disruption complicated with periodontitis by light-dark cycle control and periodontal ligature. Data are represented as the mean ± SD (*n*=3). ns, no significance; **P* < 0.05; ***P* < 0.01; ****P* < 0.001. Abbreviations: NN, mice under normal light-dark cycles without ligature; NL, mice under normal light-dark cycles with ligature; DN, mice under disturbed light-dark cycles without ligature; DL, mice under disturbed light-dark cycles with ligature; ZT, zeitgeber time.

**
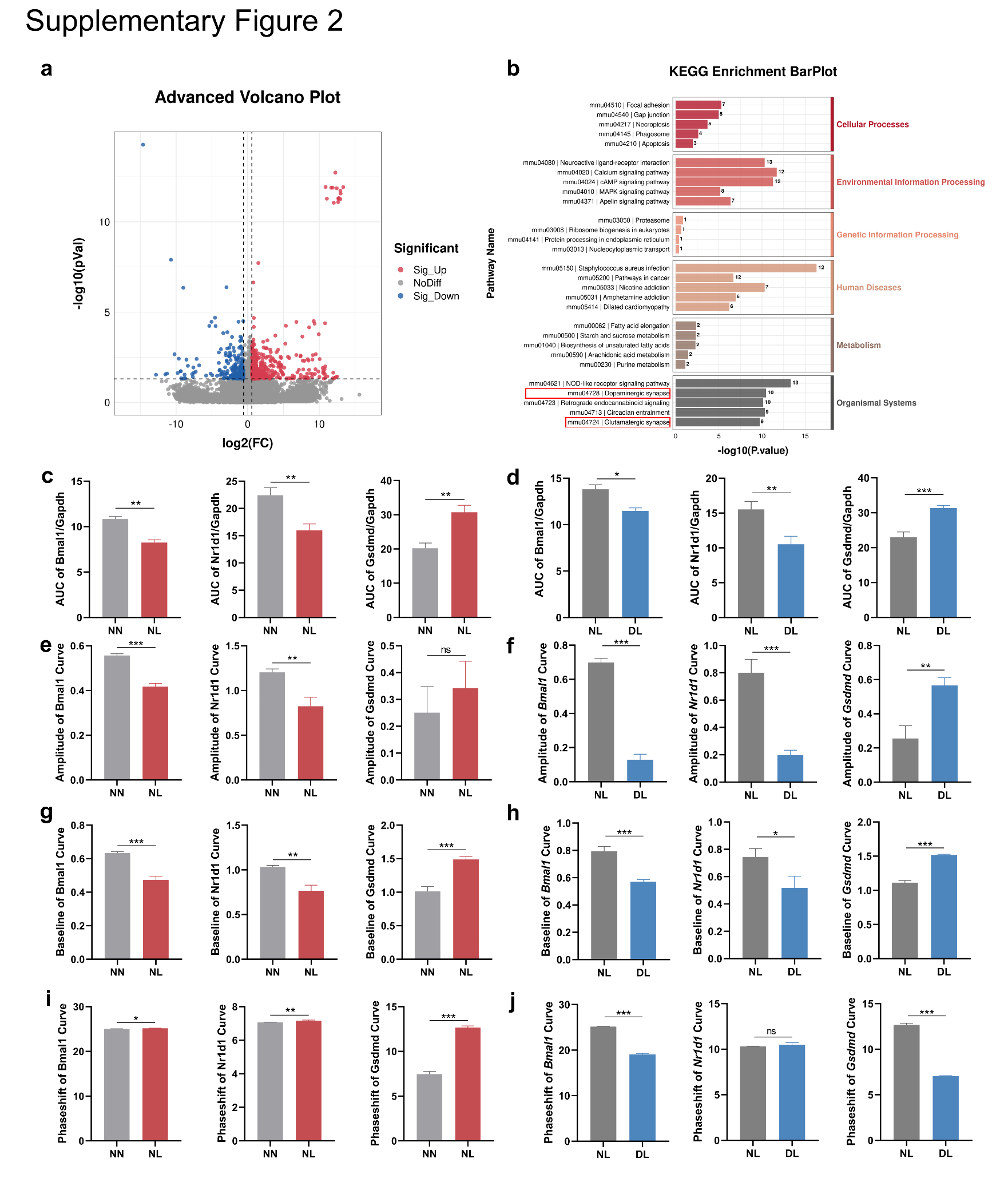
Supplementary Figure 2.** Screening of key differential genes in mouse gingiva under normal or disturbed circadian rhythm conditions. **(a)** Volcano plot for differentially expressed genes in indicated groups. **(b)** KEGG enrichment of the differential genes in inflammatory gingiva of mice under normal or disrupted circadian rhythm conditions. Quantification of the area under the cosine curves **(c, d)** as well as the amplitude **(e, f)**, the baseline **(g, h)**, and the phase shift **(i, j)** of the cosine curves fitted to the diurnal mRNA expression for *Bmal1*, *Nr1d1*, and *Gsdmd* in mouse gingiva in indicated groups. Data are represented as the mean ± SD (*n*=3). ns, no significance; **P* < 0.05; ***P* < 0.01; ****P* < 0.001. Abbreviations: NN, mice under normal light-dark cycles without ligature; NL, mice under normal light-dark cycles with ligature; DL, mice under disturbed light-dark cycles with ligature.


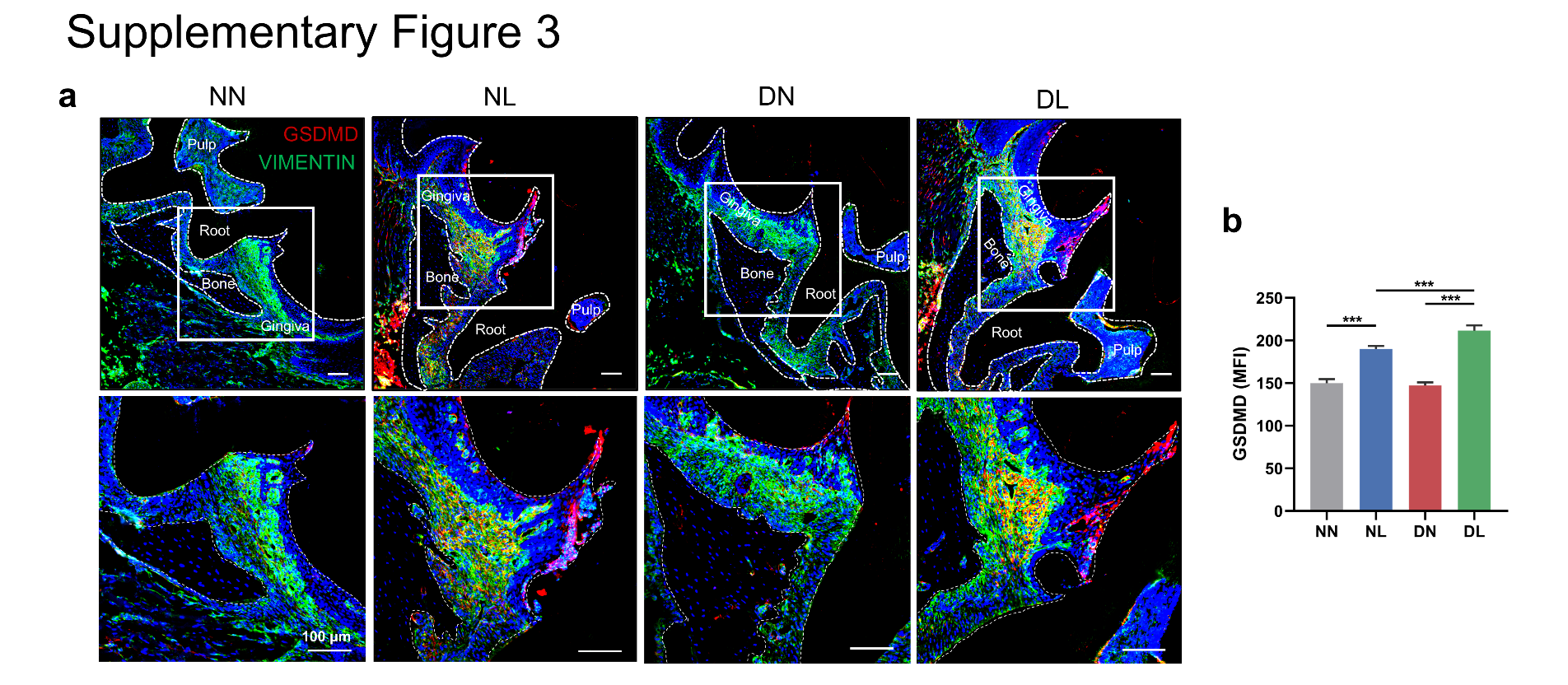


**Supplementary Figure 3.** Gingival fibroblasts are closely associated with GSDMD-mediated pyroptosis in periodontitis. **(a)** Representative images (scale bar: 100 μm) and **(b)** quantitative analysis of GSDMD expression in indicated groups using immunofluorescence staining (*n*=4). Data are represented as the mean ± SD. ****P* < 0.001. Abbreviations: NN, mice under normal light-dark cycles without ligature; NL, mice under normal light-dark cycles with ligature; DN, mice under disturbed light-dark cycles without ligature; DL, mice under disturbed light-dark cycles with ligature.


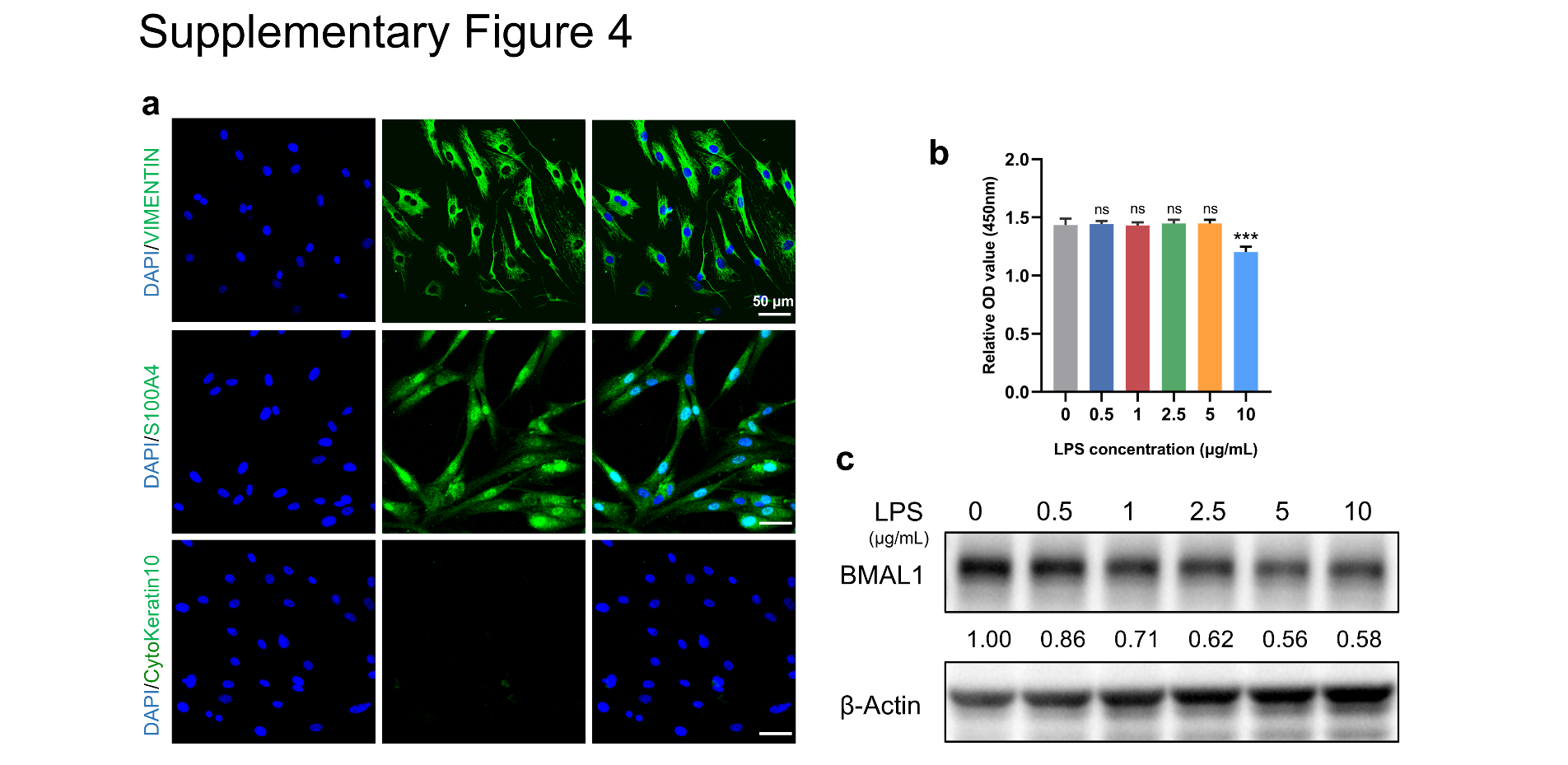


**Supplementary Figure 4.** Characterization and treatment of primary mouse gingival fibroblasts. **(a)** Representative images (scale bar: 50 μm) of VIMENTIN, S100A4, and Cytokeratin 10 staining in primary mGFs captured by LCFM. **(b)** The proliferative capacity of mGFs treated with the different concentration of *P. gingivalis* LPS for 48 h measured by CCK8 assay (*n*=5). **(c)** Relative protein expression of BMAL1 in mGFs treated with the different concentration of *P. gingivalis* LPS for 48 h measured by western blot. Data are represented as the mean ± SD. ns, no significance; ****P* < 0.001. Abbreviations: mGFs, mouse gingival fibroblasts; LPS, lipopolysaccharide; LCFM, laser confocal fluorescence microscopy; CCK8, cell counting kit-8.


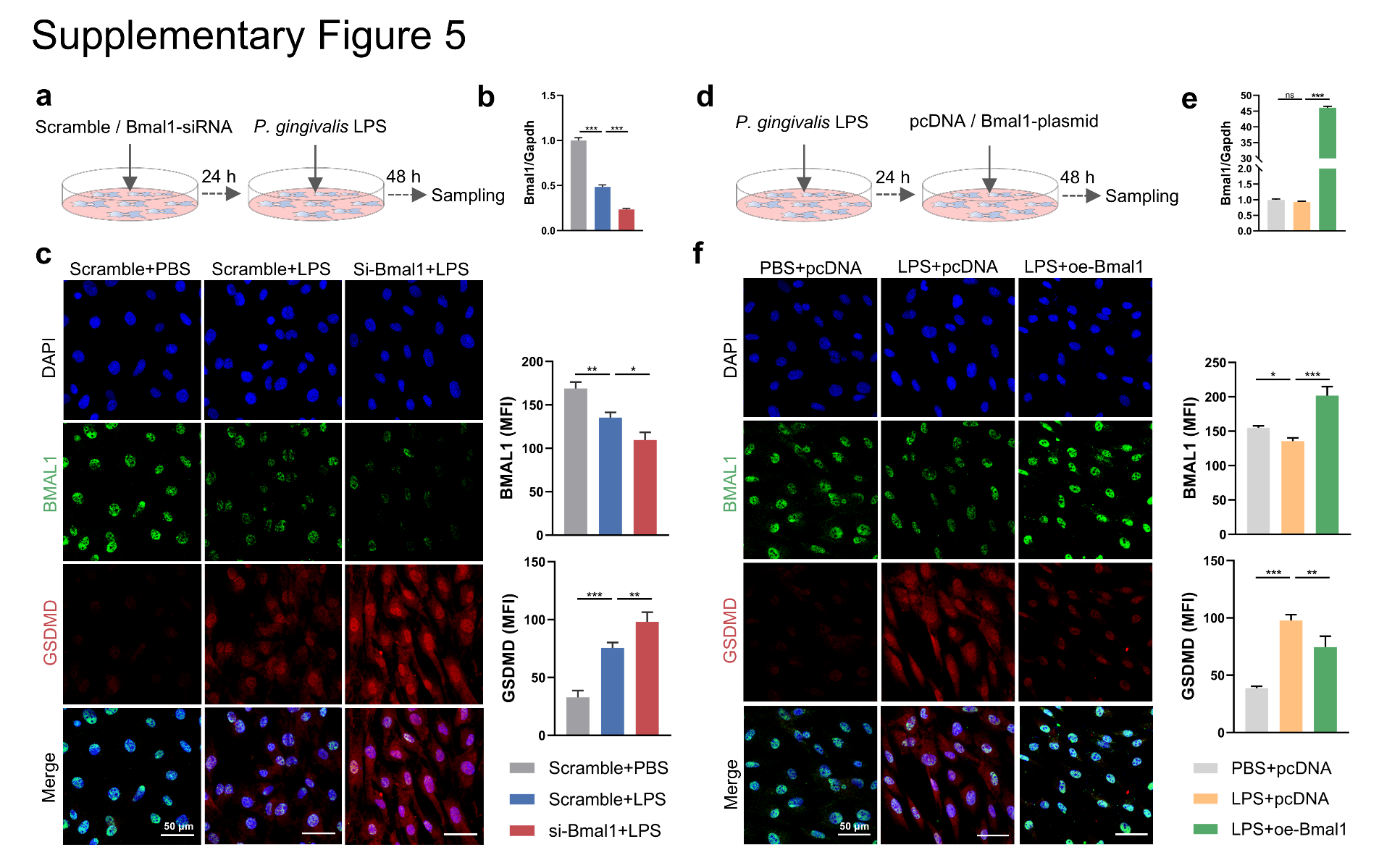


**Supplementary Figure 5.** BMAL1 plays a role in regulating the expression of GSDMD. **(a, d)** Experimental design for transfection of *Bmal1* siRNA or overexpression plasmid in *P. gingivalis* LPS-treated mGFs. **(b, e)** Verification of the efficiency of *Bmal1* interference or overexpression by qRT-PCR (*n*=3). **(c, f)** Representative images (scale bar: 50 μm) and quantitative analysis of BMAL1 and GSDMD expression in indicated groups measured by immunofluorescence staining (*n*=3). Data are represented as the mean ± SD. **P* < 0.05; ***P* < 0.01; ****P* < 0.001. Abbreviations: LPS, lipopolysaccharide; mGFs, mouse gingival fibroblasts.

**Supplementary Figure 6.** Effect of SR8278 on the expressions of BMAL1 and GSDMD in *P. gingivalis* LPS-treated mGFs. **(a)** The schema of *P. gingivalis* LPS and SR8278 treatment in mGFs. **(b)** Representative images (scale bar: 50 μm) and quantitative analysis of BMAL1 and GSDMD expression in indicated groups by immunofluorescence staining (*n*=3). Data are represented as the mean ± SD. ***P* < 0.01; ****P* < 0.001. Abbreviations: LPS, lipopolysaccharide; mGFs, mouse gingival fibroblasts.


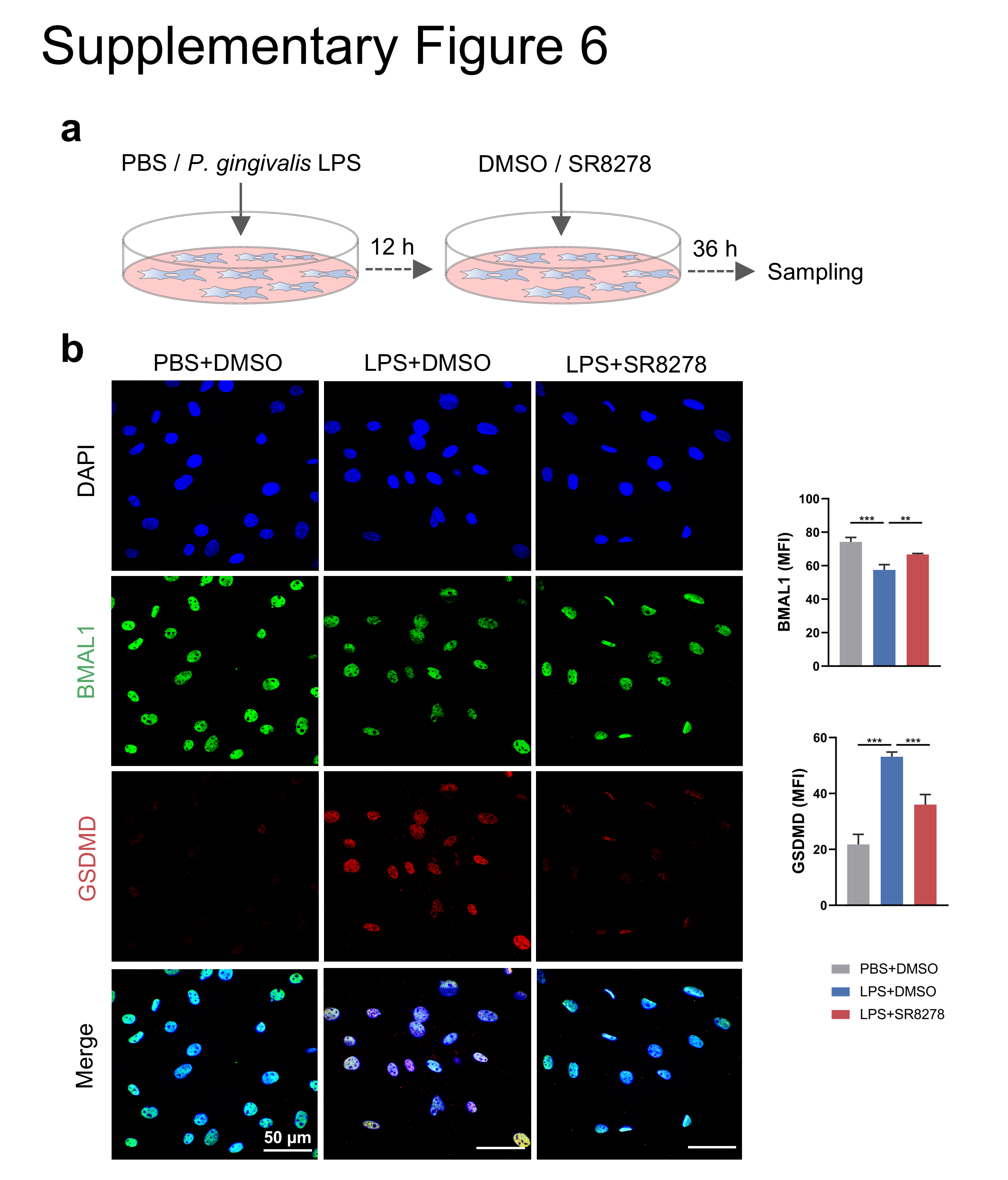

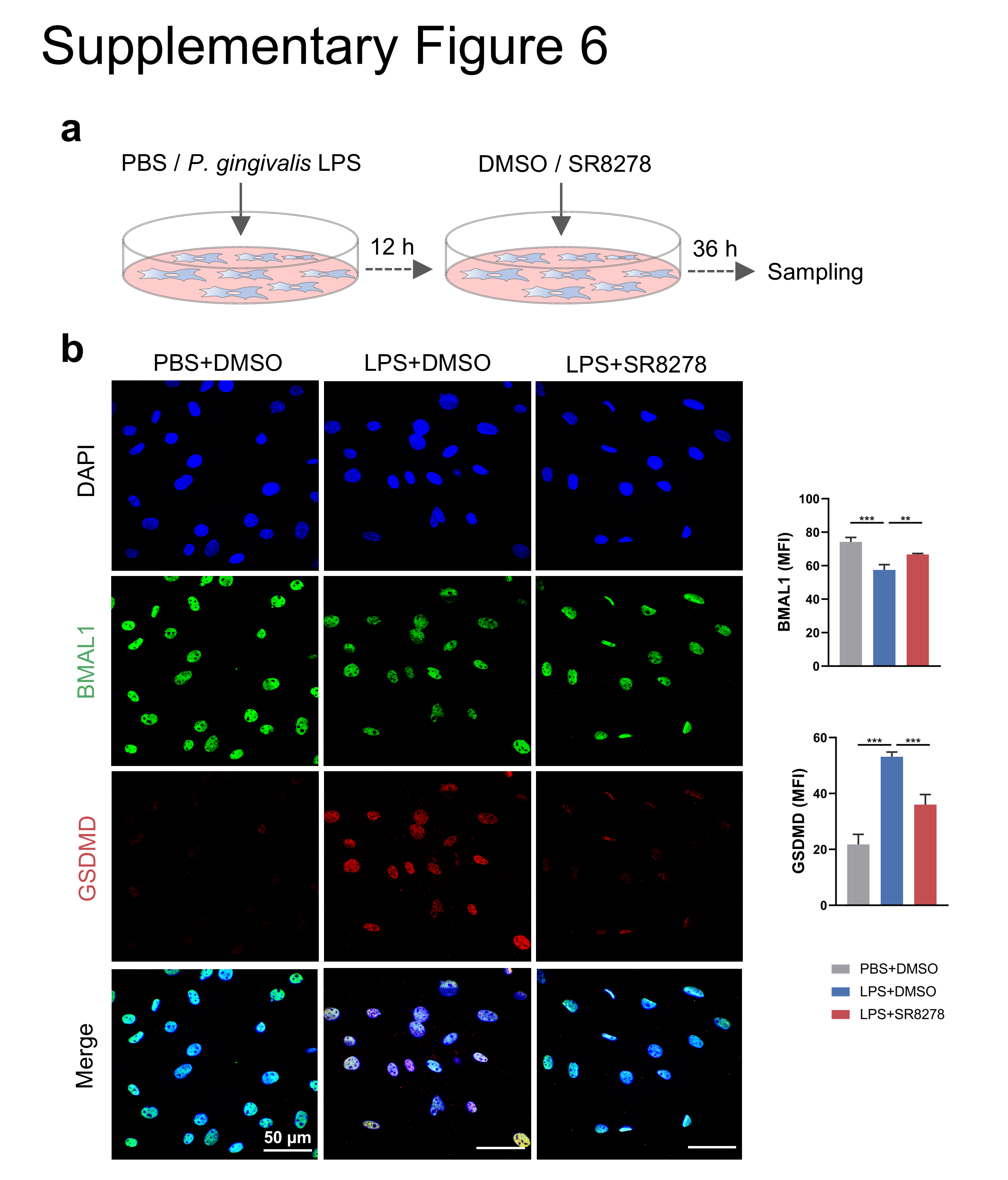


**Supplementary Figure 7.** Levels of pyroptosis-related proteins and inflammatory factors raised in periodontitis tissues. Representative images (scale bar: 50 μm) and quantitative analysis of IL-1β, IL-18, TNF-α, and N-GSDMD in the gingiva of patients with or without periodontitis measured by immunostaining (*n*=3). Data are represented as the mean ± SD. **P* < 0.05; ***P* < 0.01. Abbreviations: N-GSDMD, N-terminal fragments of GSDMD.
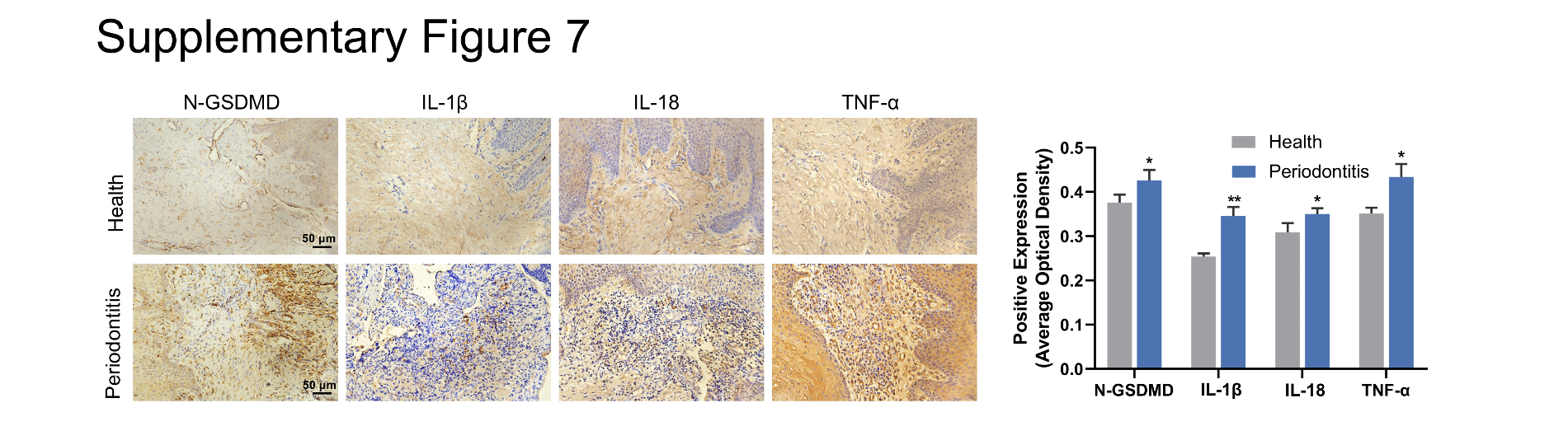


**
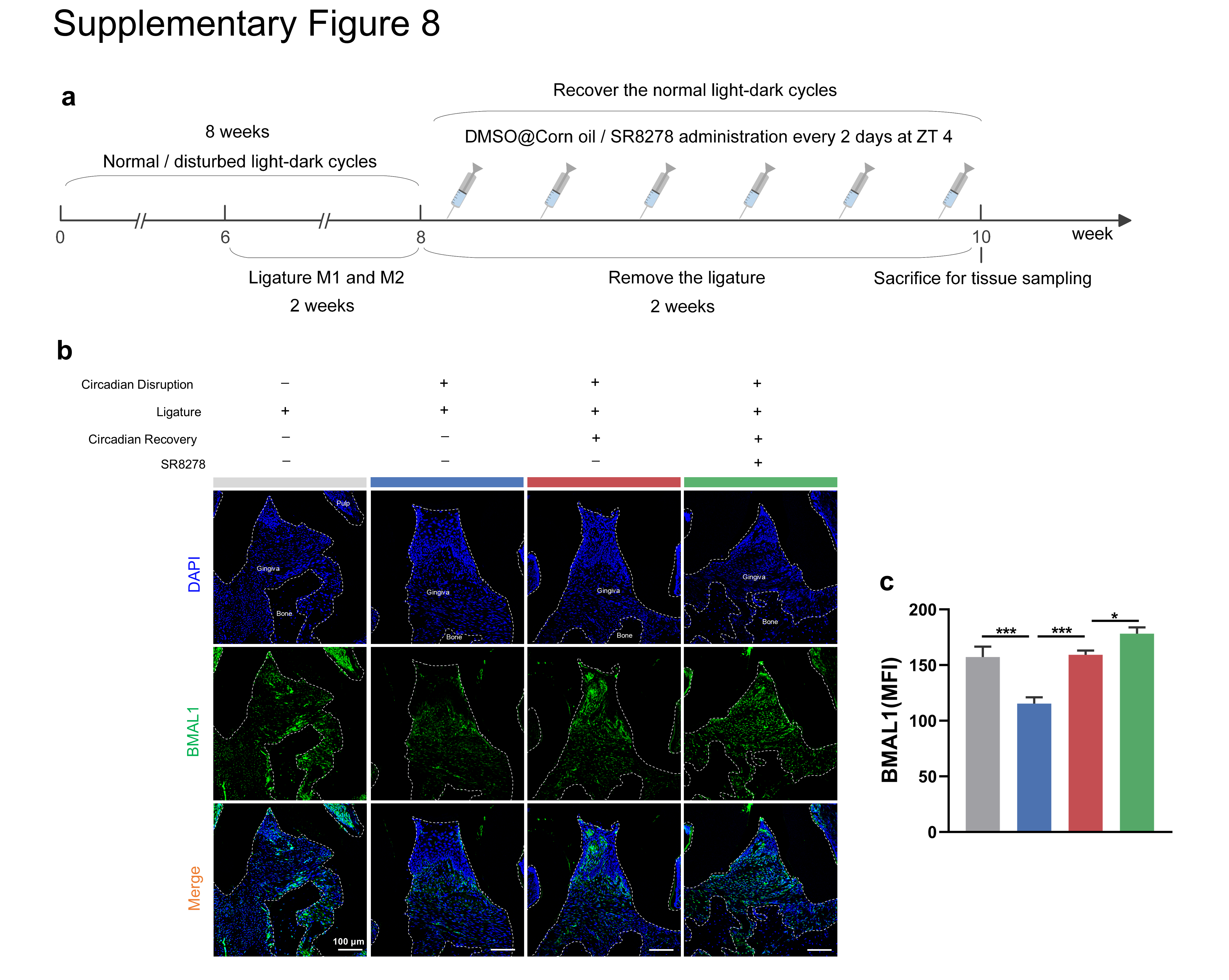
Supplementary Figure 8.** Circadian recovery and SR8278 upregulates the level of BMAL1. **(a)** Experimental design for circadian recovery model establishment in mice. **(b)** Representative images (scale bar: 100 μm) and **(b)** quantification of BMAL1 expression in indicated groups by immunofluorescence staining (*n*=3). Data are represented as the mean ± SD. **P* < 0.05; ****P* < 0.001; Abbreviations: ZT, zeitgeber time.

**Supplementary tables**

Table S1. Primer sequences used for qRT-PCR (*Mus musculus*)

| Gene | Forward primer (5’→3’) | Reverse primer (5’→3’) |
| --- | --- | --- |
| *Gapdh* | TGTGTCCGTCGTGGATCTGA | TTGCTGTTGAAGTCGCAGGAG |
| *Bmal1* | GGCTGTTCAGCACATGAAAAC | GCTGCCCTGAGAATTAGGTGTT |
| *Clock* | CTTCCTGGTAACGCGAGAAAG | GTCGAATCTCACTAGCATCTGAC |
| *Cry1* | CACTGGTTCCGAAAGGGACTC | CTGAAGCAAAAATCGCCACCT |
| *Per2* | GAAAGCTGTCACCACCATAGAA | AACTCGCACTTCCTTTTCAGG |
| *Nr1d1* | TTTTTCGCCGGAGCATCCAA | ATCTCGGCAAGCATCCGTTG |
| *Nlrp3* | ATCAACAGGCGAGACCTCTG | GTCCTCCTGGCATACCATAGA |
| *Caspase1* | CTTGGAGACATCCTGTCAGGG | AGTCACAAGACCAGGCATATTCT |
| *Gsdmd* | TTCCAGTGCCTCCATGAATGT | GCTGTGGACCTCAGTGATCT |
| *Il1b* | GAAATGCCACCTTTTGACAGTG | TGGATGCTCTCATCAGGACAG |
| *Il18* | GACTCTTGCGTCAACTTCAAGG | CAGGCTGTCTTTTGTCAACGA |

Table S2. Primer sequences used for ChIP (*Mus musculus*)

| Primer | Forward (5’→3’) | Reverse (5’→3’) |
| --- | --- | --- |
| *Gsdmd* ChIP (-1059~-906) | ATAGGGGGCTTTCTTGCCTA | TTAACCCCTGCACCATCTCT |
| *Gsdmd* ChIP (-834~-687) | GCAGACAAAACACTCCCACA | GGGTTGTGGCTATCAATGGT |
| *Gsdmd* ChIP (-699~-504) | ATAGCCACAACCCCAAGATG | TCTGCTGGCTGACATCCTAA |

Table S3. Construction information for overexpression plasmid (*Mus musculus*)

| Gene | Sequence (5’→3’) | Vector |
| --- | --- | --- |
| *Bmal1* | >NM_007489.4:521-2401 Mus musculus aryl hydrocarbon receptor nuclear translocator-like (Arntl), transcript variant 1, mRNA  ATGGCGGACCAGAGAATGGACATTTCCTCAACCATCAGCGACTTCATGTCTCCGGGCCCCACCGACCTACTCTCCGGTTCCCTGGGCACCAGTGGTGTGGACTGCAATCGCAAGAGGAAAGGCAGTGCCACTGACTACCAAGAAAGTATGGACACAGACAAAGATGACCCTCATGGAAGGTTAGAATATGCAGAACACCAAGGAAGGATCAAGAATGCAAGGGAGGCCCACAGTCAGATTGAAAAGAGGCGTCGGGACAAAATGAACAGTTTCATTGATGAATTGGCTTCTTTGGTACCAACATGCAATGCAATGTCCAGGAAGTTAGATAAACTCACCGTGCTAAGGATGGCTGTTCAGCACATGAAAACTTTGAGAGGTGCCACCAACCCATACACAGAAGCAAACTACAAGCCAACATTTCTATCAGATGACGAACTGAAACACCTAATTCTCAGGGCAGCAGATGGATTTTTGTTTGTCGTAGGATGTGACCGAGGGAAGATCCTCTTTGTCTCCGAGTCTGTCTTCAAGATCCTCAATTATAGCCAGAATGACCTTATTGGCCAGAGCTTGTTTGACTACCTGCATCCAAAAGATATTGCCAAAGTTAAGGAACAGCTATCTTCCTCGGACACTGCGCCCCGGGAGCGACTCATTGATGCCAAGACTGGACTTCCGGTTAAAACGGATATAACCCCTGGGCCCTCCCGGCTATGCTCTGGAGCCCGCCGCTCTTTCTTCTGTAGAATGAAGTGCAACAGGCCTTCAGTAAAGGTGGAAGATAAGGACTTCGCCTCTACCTGTTCAAAGAAAAAAGCAGATCGAAAAAGCTTCTGCACAATCCACAGCACAGGCTATTTGAAAAGCTGGCCACCCACGAAGATGGGGCTGGACGAAGACAATGAGCCAGACAACGAGGGCTGCAACCTCAGCTGCCTCGTTGCAATCGGGCGCCTGCACTCGCACATGGTTCCACAACCAGCGAACGGGGAAATACGGGTGAAATCTATGGAGTACGTTTCTCGACACGCAATAGATGGGAAATTTGTTTTTGTAGATCAGAGGGCGACAGCTATTTTGGCGTATCTACCACAGGAACTTCTAGGTACATCATGTTATGAGTATTTTCATCAAGACGACATAGGACACCTCGCAGAATGTCACAGGCAAGTTTTACAGACAAGAGAAAAGATCACAACTAATTGCTATAAGTTTAAGATCAAAGATGGTTCTTTTATCACGCTACGAAGTCGATGGTTCAGTTTCATGAACCCGTGGACCAAGGAAGTTGAATACATTGTCTCAACCAACACTGTTGTTTTAGCCAATGTCCTGGAAGGCGGGGACCCAACCTTCCCGCAGCTAACAGCACCCCCCCACAGCATGGACAGCATGCTGCCCTCTGGAGAAGGTGGCCCAAAGAGGACTCATCCCACTGTCCCAGGCATTCCAGGGGGAACCAGAGCCGGAGCAGGAAAAATAGGTCGAATGATCGCGGAGGAAATCATGGAAATCCACAGGATAAGAGGGTCATCGCCTTCCAGCTGTGGCTCCAGCCCGCTGAACATCACAAGTACGCCTCCCCCTGATGCCTCTTCTCCAGGAGGCAAGAAGATTCTAAATGGAGGGACTCCAGACATTCCTTCCACTGGACTATTACCAGGGCAGGCTCAGGAGACCCCAGGGTATCCCTATTCTGATAGTTCTTCTATTCTTGGTGAGAACCCCCACATAGGCATCGATATGATAGATAACGACCAAGGATCAAGTAGTCCCAGTAACGATGAGGCAGCAATGGCTGTCATCATGAGCCTCTTGGAAGCAGATGCGGGGCTGGGTGGCCCCGTTGACTTTAGTGACTTGCCATGGCCGCTGTAG | pEX-3 (pGCMV/MCS/Neo) |
